# The utility of the ‘Arable Weeds and Management in Europe’ database: Challenges and opportunities of combining weed survey data at a European scale

**DOI:** 10.1111/wre.12562

**Published:** 2022-12-08

**Authors:** Helen Metcalfe, Jana Bürger, Christoph von Redwitz, Alicia Cirujeda, Silvia Fogliatto, Denise F. Dostatny, Bärbel Gerowitt, Michael Glemnitz, José L. González‐Andújar, Eva Hernández Plaza, Jordi Izquierdo, Michaela Kolářová, Jevgenija Ņečajeva, Sandrine Petit, Gyula Pinke, Matthias Schumacher, Lena Ulber, Francesco Vidotto, Guillaume Fried

**Affiliations:** ^1^ Net Zero and Resilient Farming, Rothamsted Research, West Common Harpenden Hertfordshire UK; ^2^ Crop Health, Faculty of Agricultural and Environmental Sciences University of Rostock Rostock Germany; ^3^ Institute for Plant Protection in Field Crops and Grassland, Julius Kuehn‐Institut (JKI), Federal Research Centre for Cultivated Plants Braunschweig Germany; ^4^ Sistemas Agrícolas, Forestales y Medio Ambiente, Plant Protection, Agrifood Research and Technology Centre of Aragón (CITA) Zaragoza Spain; ^5^ Department of Agricultural, Forest and Food Sciences (DISAFA) University of Torino Grugliasco Italy; ^6^ Plant Breeding and Acclimatization Institute – National Research Institute, National Centre for Plant Genetic Resources Błonie Poland; ^7^ Cultivar Testing, Nursery and Genebank Resources Department National Institute for Horticultural Research Skierniewice Poland; ^8^ Provision of Biodiversity in Agrarian Systems Research Area II: “Land Use and Governance”, Leibniz Centre for Agricultural Landscape Research (ZALF) Müncheberg Müncheberg Germany; ^9^ Instituto de Agricultura Sostenible, Spanish National Research Council (CSIC) Córdoba Spain; ^10^ Department of Plant Protection National Institute for Agricultural and Food Research and Technology, Spanish National Research Council (INIA‐CSIC) Madrid Spain; ^11^ Department of Agrifood Engineering and Biotechnology Politechnical University of Catalunya Castelldefels Spain; ^12^ Department of Agroecology and Crop Production, Faculty of Agrobiology, Food and Natural Resources Czech University of Life Sciences Prague Praha – Suchdol Czech Republic; ^13^ Institute for Plant Protection Research ‘Agrihorts’ Latvia University of Life Sciences and Technologies Jelgava Latvia; ^14^ Agroécologie, AgroSup Dijon, INRAE Université de Bourgogne Franche‐Comté Dijon Cedex France; ^15^ Faculty of Agricultural and Food Sciences Széchenyi István University Mosonmagyaróvár Hungary; ^16^ Department of Weed Science, Institute of Phytomedicine, Faculty of Agricultural Sciences University of Hohenheim Stuttgart Germany; ^17^ Plant Health Laboratory Anses Montferrier‐sur‐Lez France

**Keywords:** abundance measures, arable plants, cover estimates, data collection, management, nomenclature, plot size, sampling bias, weed community, weeds

## Abstract

Over the last 30 years, many studies have surveyed weed vegetation on arable land. The ‘Arable Weeds and Management in Europe’ (AWME) database is a collection of 36 of these surveys and the associated management data. Here, we review the challenges associated with combining disparate datasets and explore some of the opportunities for future research that present themselves thanks to the AWME database. We present three case studies repeating previously published national scale analyses with data from a larger spatial extent. The case studies, originally done in France, Germany and the UK, explore various aspects of weed ecology (community composition, management and environmental effects and within‐field distributions) and use a range of statistical techniques (canonical correspondence analysis, redundancy analysis and generalised linear mixed models) to demonstrate the utility and versatility of the AWME database. We demonstrate that (i) the standardisation of abundance data to a common measure, before the analysis of the combined dataset, has little impact on the outcome of the analyses, (ii) the increased extent of environmental or management gradients allows for greater confidence in conclusions and (iii) the main conclusions of analyses done at different spatial scales remain consistent. These case studies demonstrate the utility of a Europe‐wide weed survey database, for clarifying or extending results obtained from studies at smaller scales. This Europe‐wide data collection offers many more opportunities for analysis that could not be addressed in smaller datasets; including questions about the effects of climate change, macro‐ecological and biogeographical issues related to weed diversity as well as the dominance or rarity of specific weeds in Europe.

## 1 INTRODUCTION

Weed vegetation surveys are commonly used in weed science to assess the impacts of agricultural practices on weed flora (e.g., Pinke et al., 2012), determine causes of yield losses (e.g., Adeux et al., 2019), monitor conservation efforts (e.g., Kolářová et al., 2013) and map species distributions (e.g., Hanzlik & Gerowitt, 2012). Many surveys are designed for a particular purpose and so the collected data, and associated metadata, are specific to the particular research question. As such, the resulting local databases can contain different methodologies and data types (Bürger et al., 2022). Despite such discrepancies, there is potential for added benefits when surveys are analysed in combination, as demonstrated by the success of several plot‐vegetation databases (e.g., European Vegetation Archive [EVA] [Chytrý et al., 2016], European Weed Vegetation Database [Küzmič et al., 2020]). The Arable Weeds and Management in Europe (AWME) database (Bürger et al., 2020) provides a new resource for combining arable weed survey data and complementary management information from across Europe.

The value of combining data or findings from multiple studies is clear, as illustrated by several meta‐analyses aiming at improving our understanding of the response of weed communities to drivers of change (e.g., Gu et al., 2021; Richner et al., 2015). Meta‐analyses use previously published results to understand the net effect of a specific driver on weed community response. However, the utility of this approach is limited by the accessibility of published statistical results and appropriate measures of confidence in published articles. There is, therefore, scope to provide more robust analyses by returning to the raw data, as demonstrated by the success of similar data collections in adjacent scientific disciplines (e.g., CESTES database [Jeliazkov et al., 2020], which is dedicated to analyses at the metacommunity level including species traits). However, the key challenges facing analysts using the AWME database (Table 1) has not been widely explored.

**TABLE 1:** Key challenges associated with the analysis of data coming from multiple weed vegetation surveys and potential solutions that are incorporated within the Arable Weeds and Management database and our analyses.

Challenge	Potential problems	How it is addressed within AWME	How we address it in our analysis
(i) Species nomenclature	Plant taxonomy changes rapidly and inconsistent species nomenclature can reduce the usefulness of a vegetation database	Records from all surveys are standardised to a common reference frame (EURO+MED, 2006).	No additional measures required
(ii) Comparability of environment and management data	Surveys collected for different purposes may have varying levels of detail for environmental and management practices	Management variables are grouped into broad categories (e.g., tillage, crop, previous crop and herbicide treatment) with the raw data maintained alongside. Data are also supplemented for completeness with, for example, SoilGrids (de Sousa et al., 2020) and WorldClim (Fick & Hijmans, 2017).	Included only the derived variables in analysis to retain sufficient detail for analysis whilst allowing a larger number of surveys to be considered
(iii) Compatibility of abundance measures	In vegetation surveys, a range of measures of abundance are used including counts of individuals and visual assessments of cover on both continuous and discrete scales.	Relevant information on abundance measures used in each survey is provided to encourage careful interpretation of any analyses performed on data recorded using different measures (see Anderson et al., 2012).	Converted observations to a common scale (presence/absence [FR] or Barralis scale [DE]) or standardised abundance measures within each survey to zero mean and unit variance (UK). Transformations lead to some loss of information but allow all records to be retained in the analysis.
(iv) The relative timing (season) of sampling	Plant community composition is temporally dynamic, whilst surveys represent a snapshot.	Relevant information on timing of each survey is provided. Users are encouraged to focus on the timing of the survey relative to the crop phenology rather than the absolute date.	Considered in the analysis directly (FR) or indirectly via using the survey as a random effect (UK).
(v) Balance of data and spatial sampling biases	Imbalance in the relative size of datasets can introduce biases. Some datasets may be small in sample number and spatial extent, whilst others cover a wider geographic or temporal extent.	Not explicitly addressed within AWME as it may or may not present a problem for a specific analysis	Randomly selected one observation from fields with multiple records (FR) Trialled a spatial subsampling procedure (DE) and fitted variograms to the response variables to test for spatial autocorrelation (UK).
(vi) Disparity in plot size between surveys	Plot sizes vary according to the purpose of the survey, and available resources. Observations on larger plots will likely have higher species richness, and the co‐occurrence of species may depend on plot size (Chytrý & Otýpková, 2003).	Information about survey methodology and plot size are included within AWME so that the user may make an informed decision as to which approach best suits their needs.	Retained all plot sizes in analysis (FR and DE) as previous work has shown that analysing data from varying plot sizes does not introduce strong bias (Peterka et al., 2020) Accounted for differences between surveys (not just plot size) by including the survey as a random effect (UK).

Here, we will explore the utility of the AWME data collection using three case studies. In each case study, we will focus on an analysis previously published using data from a national scale weed survey (each of which is a component dataset within the AWME database). Each of the original studies posed a different question about weed communities and their ecology. Using a three‐stage process we will determine whether (a) the data contained within AWME allow this question to be addressed at the European scale, (b) it is possible to overcome key challenges associated with analysing data gathered for different purposes and (c) the conclusions of the original publication and our reanalysis remain consistent across scales. Through this work, we aim to examine the utility of the AWME data collection for European scale analysis and guide future analysts in the best practice for using the AWME data collection to avoid key challenges associated with data collections of this kind.

## 2 MATERIALS AND METHODS

### 2.1 The AWME database

In 2019, the working group Weeds and Biodiversity of the European Weed Research Society set out to form a data collection of primary arable weed vegetation records. The resulting AWME (Bürger et al., 2020) database currently comprises 36 surveys of arable weed vegetation conducted between 1996 and 2018 across 12 countries (Tables S1 and S2) and contains >40 000 observations of weed vegetation. The unifying feature of these records is that each consists of a list of species found on a plot of a specific size from an arable field or its margin. Observations are complemented by metadata including the survey date and geographic coordinates. The distinguishing feature of the AWME database (from other collections such as EVA) is that observations are supplemented with information on the agricultural management of the survey site, making the data more useful to agronomists and weed scientists seeking to understand the impact of agricultural practices on weed communities. These agricultural management data include information on crop, previous crops and rotations, tillage or weed control treatments. The 36 included weed surveys were conducted using different combinations of sampling design and methodology with varying levels of complexity. Whilst some studies had a narrow focus on a single crop, study region, or growing season, other studies were designed at a national scale sampling many crops and/or growing seasons. Different surveys focussed on distinct parts of the field, for example, to study edge effects or to compare plots with and without herbicide treatments. Most of the surveys in AWME were done just before crop harvest, when most weed species were mature, however others focussed on earlier crop growth stages concentrating on significant time points for weed management decision‐making. Various abundance measures (e.g., counts, cover) were used to record species occurrence. These differences between surveys give rise to several key challenges for analysis. Many of these are addressed directly within AWME, whilst others were important to consider during our analyses (Table 1).

### 2.2 The case studies

We selected three case studies where analyses had previously been published posing a range of ecological questions regarding weed communities. The original publications interrogated a national scale weed survey, and each of these national scale surveys are now included within the AWME data collection. Our first case study (FR) examined the important environmental and management factors determining weed species composition in France. The original publication by Fried et al. (2008) used data from a French national weed survey (Biovigilance Flore). The second case study (DE) was a more focussed study excluding the effect of crop type by focussing on maize crops to understand the role of the environment and management in weed community composition. In their original analysis published in 2015, de Mol et al. interrogated weed survey data from German maize crops. Our third case study (UK) follows Metcalfe et al. (2019) who used data from a national weed survey in the UK (Farm Scale Evaluations, Heard et al., 2003) to study the effect of landscape features, environment and management on weed diversity and abundance in arable fields.

### 2.3 Analysis

To recreate the case studies, we kept the analytical methods as true to each of the original publications as possible.

We used canonical correspondence analyses (CCA; ter Braak, 1986) for the FR case study. Following Lososová et al. (2004), we tested for gross and net effects of each explanatory variable on weed species composition. The explanatory variable considered were latitude (°N), longitude (°E), mean annual temperature (°C), annual precipitation (mm), soil pH, crop type, previous crop, herbicide treatment (presence/absence), position in the field (core/edge), sampling season and year of sampling. Separate CCAs with a single explanatory variable were used to test gross effects. The effect of a particular variable after partitioning out the effect shared with the other explanatory variables (i.e., net effect) was tested using partial CCAs (pCCA), each with a single explanatory variable and the other 10 (9) variables used as covariates (see Step 1 below for details of explanatory variables). Significances were tested by 1000 permutation tests. We used the ratio of a particular canonical eigenvalue over the sum of all eigenvalues (total inertia) as a measure of the proportion of variation explained by each factor. We used the cca() function from the vegan package in R (Oksanen et al., 2022) to calculate the CCA.

In the DE case study, we compare the combined effect of three groups of variables on weed species composition in the DE case. We separated the effects of environment (latitude [°N], longitude [°E], precipitation in summer [mm], minimum temperature [°C], mean temperature [°C], phosphor content, nitrogen content, pH), management (previous crop, tillage type [ploughing present/absent]), and the year on weed species composition using variance partitioning based on redundancy analysis (RDA; Legendre, 2008). Similar to the approach for the FR case, variance partitioning calculates gross and net effects. In this case, gross effect was calculated by using all variables of a group and the net effect by using the variables of the other groups of covariates. Each of the groups were formed by different explanatory variables. For the variation partitioning, we used Hellinger transformation to avoid horseshoe effects and to reduce the weight of rare species (Legendre & Gallagher, 2001). We used 500 permutations of the analysis to test for significance. In the data from AWME, there were some correlations between environmental variables. However, to keep the analysis as similar as possible to the original study we opted to retain all variables in our analyses. We used the vegan package in R (Oksanen et al., 2022) to calculate the RDA and the variance partitioning respectively.

In contrast to the multivariate analyses in the FR and DE case studies, we used generalised linear mixed effects models (GLMMs) in the UK case study to investigate the effect of crop, herbicide treatment (presence/absence) and position in field (core/edge) on weed species richness and abundance. Species richness and weed abundance (obtained using count data) were assumed to follow a Poisson distribution and the rescaled data (see Table 1) were assumed to follow a normal distribution. We used the canonical link function (natural logarithm for Poisson responses, identity for normal responses). We estimated the dispersion parameter to account for over and under dispersion. We considered the following terms in the fixed effects model: position in field (core/edge), crop type, herbicide treatment (presence/absence). We also included the second and third‐order interactions between these fixed effects. Terms were selected using backwards elimination according to the largest *p*‐value given by an approximate *F*‐test when that term was dropped (Kenward & Roger, 1997). The final predictive model was chosen when all remaining terms gave significant values (*p* ≤ 0.05) for an *F* test when dropped from the model. All statistical analyses were done using R (R Core Team, 2022), the GLMMs were fitted using Genstat (Payne, 2013) to correspond with the original publication.

To address our aims objectively, we took a three‐step approach to separate the effect of any data transformations and changes in scale.

#### 2.3.1 Step 1: Reframing the scope

To address some of the challenges associated with combining disparate datasets several of the variables used in the original studies have been consolidated or simplified within AWME (Table 1) as such it was necessary to redefine the scope of each of our case studies and repeat the analysis from the original publications using only the original data in the form as it is contained within AWME. This gave us a baseline result within the original spatial extent and before any additional data transformations.

In Step 1 of case study FR, we redefined the scope of the analysis to include only 11 of the original 14 explanatory variables analysed by Fried et al. (2008) (retaining latitude [°N], longitude [°E], mean annual temperature [°C], annual precipitation [mm], soil pH, crop type, previous crop, herbicide treatment [yes/no], position in the field [core/edge; in the French data all records were taken in field cores], sampling season [spring, summer, autumn, winter] and year of sampling). We excluded minor crops represented in fewer than 100 fields and simplified the previous crop variable into five categories (autumn‐sown dicot crop, autumn‐sown monocot crop, spring‐sown dicot crop, spring‐sown monocot crop, other—including fallow and pluriannual forage crops) which characterised the legacy effect of past disturbances (Gaba et al., 2014), the sowing date influencing the timing of disturbances and the type of crop being partly related to the nature of the disturbances (types of herbicides). To reduce noise due to rare species, we only retained the 217 weed species that occurred in more than 50 fields (~1% of the analysed fields).

For step one of the DE case study, we redefined the scope of the analysis to include the previous crop, tillage (± plough), longitude (°E), latitude (°N), three climate variables (precipitation of the warmest quarter of the year [mm], minimum and mean yearly temperature [°C] from WorldClim data version 2.1 [Fick & Hijmans, 2017]), and three soil properties (phosphorus and nitrogen content, and pH from LUCAS grids [Orgiazzi et al., 2018]). This gave 10 variables for use in the RDA compared to the original work by de Mol et al. (2015) which used 14 variables in their analysis of the weeds of maize crops in Germany.

In their original study, which is the focus of our UK case study, Metcalfe et al. (2019) did repeated analyses on datasets from the same fields before and after herbicide application. However, datasets of this kind are rare in AWME and so we redefined the scope of the analysis to instead include herbicide treatment (presence/absence) as an explanatory variable. We also included crop type and field part (core/edge; in the original publication this was a continuous variable representing transects with samples at various distances from the edge).

#### 2.3.2 Step 2: Transforming the response variables

To address some of the key challenges identified in Table 1 it was necessary to transform our response variables to allow compatibility across the different survey data sets. Here, we trialled three different approaches: For FR and DE, we transformed all data to presence/absence and the Barralis (1976) scale respectively (the six Barralis classes: ‘+’, ‘1’, ‘2’, ‘3’, ‘4’ and ‘5’ represented 0.0005, 0.5, 1.5, 11.5, 35.5 and 75.5 individuals/m^2^ respectively). Whilst for the UK case study, we standardised the response variable within each survey data set to have zero mean and unit variance. We repeated the statistical analyses with the transformed response variables, again using only the data from within the original spatial extent. This step allowed us to understand better the robustness of any transformations or changes in analysis made to address the differences in abundance measure.

#### 2.3.3 Step 3: Scaling up to the European scale

**FIGURE 1:**
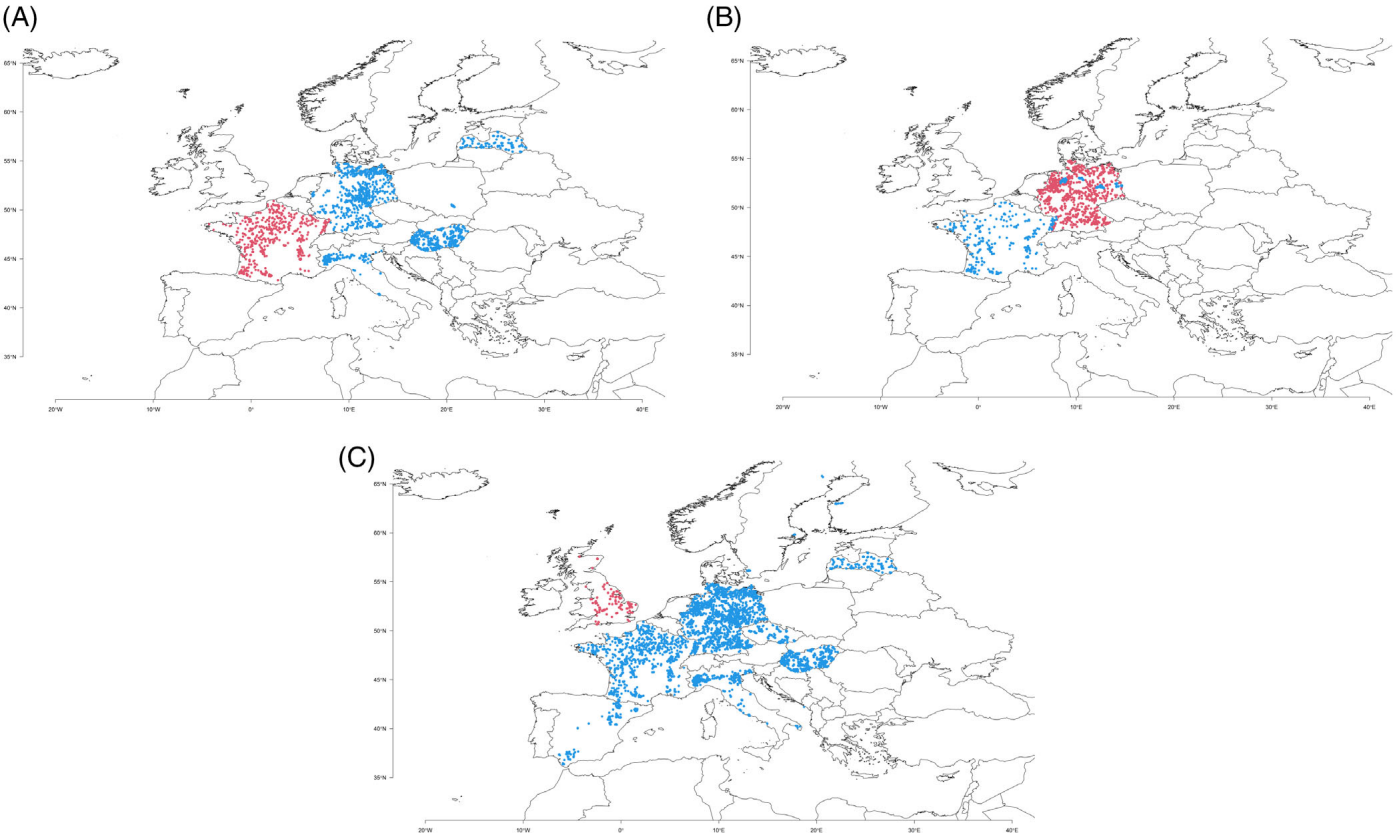
Distribution of the sample plots used in (A) case study FR, (B) case study DE and (C) case study UK. Red dots represent the fields used in the original study; blue dots represent the additional fields from the AWME

In Step 3, we again repeated each analysis with the transformed response variable used in Step 2 but at a wider spatial extent incorporating all relevant data from AWME from across Europe (Figure 1). This final stage allowed us to assess whether the changes introduced in the previous two steps leave sufficient remaining records to make data analysis possible at a continental scale and to better understand whether results obtained from analyses at a national scale are truly representative of universal concepts within weed ecology.

We avoided the issue of spatial biases in the FR case study by randomly selecting one observation from fields with multiple records across all three steps of our analysis. To account for potential spatial bias (Table 1) in the European scale analysis of the other two case studies, we additionally trialled a spatial subsampling procedure (DE, see Figure S1) and fitting variograms to the response variable to test for spatial autocorrelation (UK), however, these were found to be unnecessary and so the results are not presented here.

## 3 RESULTS

**FIGURE 2:**
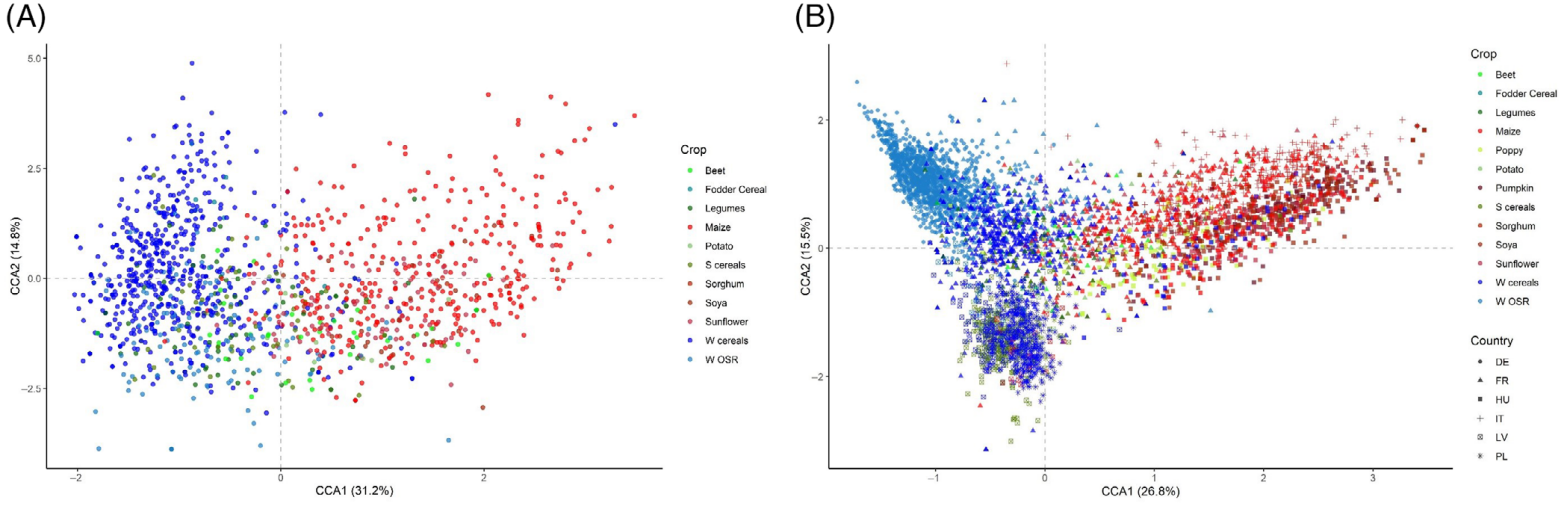
Ordination diagrams of the canonical correspondence analyses (CCA) at (A) the French scale (CCA1: 31.2% of the variation explained; CCA2: 14.8%) and (B) the European scale (CCA1: 26.8% of the variation explained; CCA2: 15.5%) with sites colour depending on crop type (blue: winter crops, green: spring crops, red: summer crops). W cereals: winter cereals, W OSR: winter oilseed rape. In panel (B), the shapes represent the country. DE, Germany; FR, France; HU, Hungary; IT, Italy; LV, Latvia; PL, Poland

In our FR case study, crop type was the top‐ranked explanatory variable in all three steps of our analysis (Table 2). The relative importance of longitude was notable at the European scale where it was the second most important explanatory variable (rank 7 at national scale). The importance of pH and precipitation at the national scale (rank 4 and 5 respectively) was less marked at the European scale (rank 8 and 7 respectively). Most of the weed community composition differences between crop types is related to differences in crop sowing date expressed on CCA axis 1, from autumn‐sown crops on negative to spring‐sown crops on positive loadings (Figure 2).

**TABLE 2:** Gross (CCA) and net (pCCA) effect of covariates on weed species composition at the European (*n* = 4686 fields) and the French level (*n* = 1396 fields)

Variables	Step 1. France (abundance)			Step 2. France (presence‐absence)			Step 3. Europe (presence‐absence)		
	Gross effect (%)	Net effect (%)	Order	Gross effect (%)	Net effect (%)	Order	Gross effect (%)	Net effect (%)	Order
Crop type	3.632	2.488	1	3.445	1.850	1	6.022	1.517	1
Longitude	0.438	0.249	7	0.423	0.200	7	1.522	0.455	2
Previous crop	1.217	0.845	3	1.187	0.591	2	1.903	0.408	3
Season	0.633	1.389	2	1.566	0.440	3	3.418	0.333	4
Temperature	0.656	0.311	5	0.765	0.201	6	1.964	0.291	5
Year	0.384	0.279	6	0.270	0.169	9	1.135	0.261	6
Precipitation	0.645	0.230	9	0.804	0.234	5	0.829	0.234	7
Soil pH	0.630	0.311	4	0.596	0.235	4	0.549	0.225	8
Latitude	0.687	0.247	8	0.862	0.193	8	1.935	0.195	9
Field Part	‐	‐	‐	‐	‐	‐	1.174	0.180	10
Treatment	0.182	0.130	10	0.134	0.139	10	1.532	0.116	11

*Note*: Variables are ordered by decreasing values of net effect at the European scale.

**FIGURE 3:**
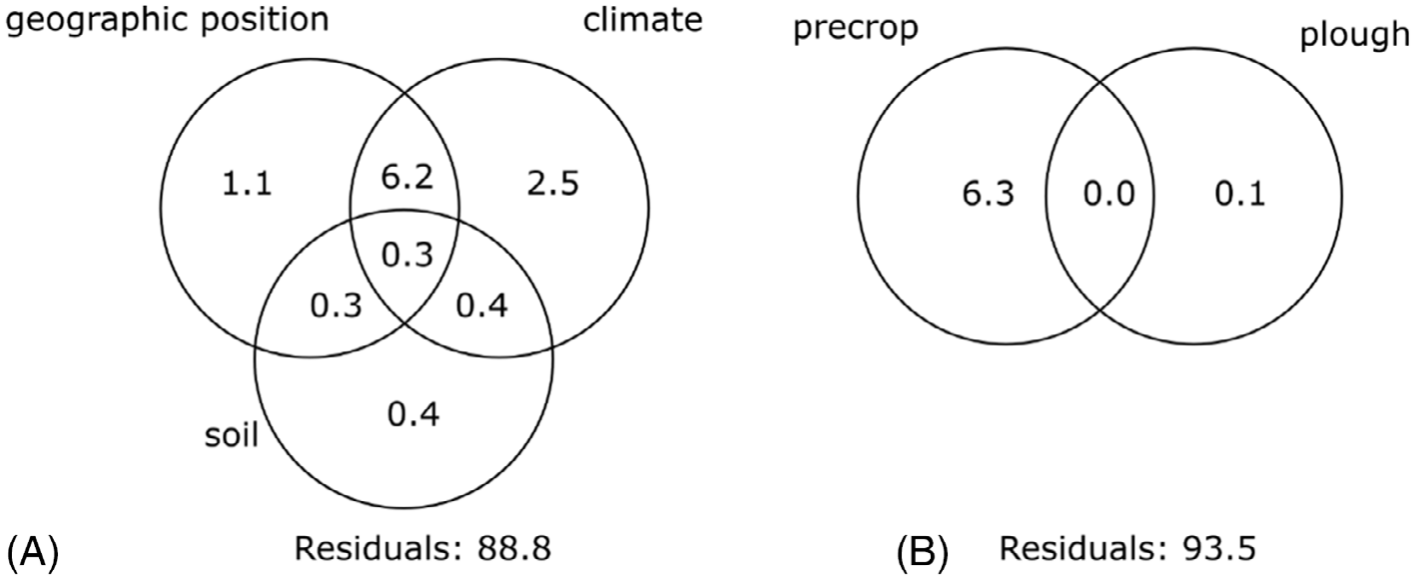
Variance partitioning for (A) environmental variables and (B) management variables. Values are the proportion of explained inertia of weed species composition in the European dataset which can be attributed to the variables. Overlapping circles show the combined effect of variables. ‘Precrop’ refers to the crop grown in the season prior to sampling

In our DE case study, the net effect of environment was lower at the European scale than at the national scale, however the combined effect of environment, management and year increased from 10.5 to 15.8 (%) for gross effect and from 0.1 to 2.4 (%) net effect (E × M × Y in Table 3). Focusing only on environmental variables (Figure 3), the combination of climate and geographical position explained most of the variation, whilst the net effects of climate, geographical position and soil were relatively small.

**TABLE 3:** Gross and net effect (variance partitioning on basis of RDA) of groups of covariates on weed species composition at the German (*n* = 2593 fields) and European level (*n* = 3355 fields) in percentage explained variance

Group of variables	Step 1. Germany (in count)		Step 2. Germany (in Barralis scale)		Step 3. Europe (in Barralis scale)	
	Gross effect (%)	Net effect (%)	Gross effect (%)	Net effect (%)	Gross effect (%)	Net effect (%)
Environmental (E)	7.9	6.5	7.8	6.4	11.2	5.6
Management (M)	2.8	1.7	2.8	1.6	6.5	1.7
Year (Y)	1.2	0.8	1.2	0.8	6.4	2.7
E × M	9.6	1.0	9.5	1.0	13.2	2.2
E × Y	8.8	0.2	8.7	0.2	14.2	1.1
M × Y	3.9	0.0	3.9	0.0	10.3	0.3
E × M × Y	10.5	0.1	10.3	0.1	15.8	2.4

**FIGURE 4:**
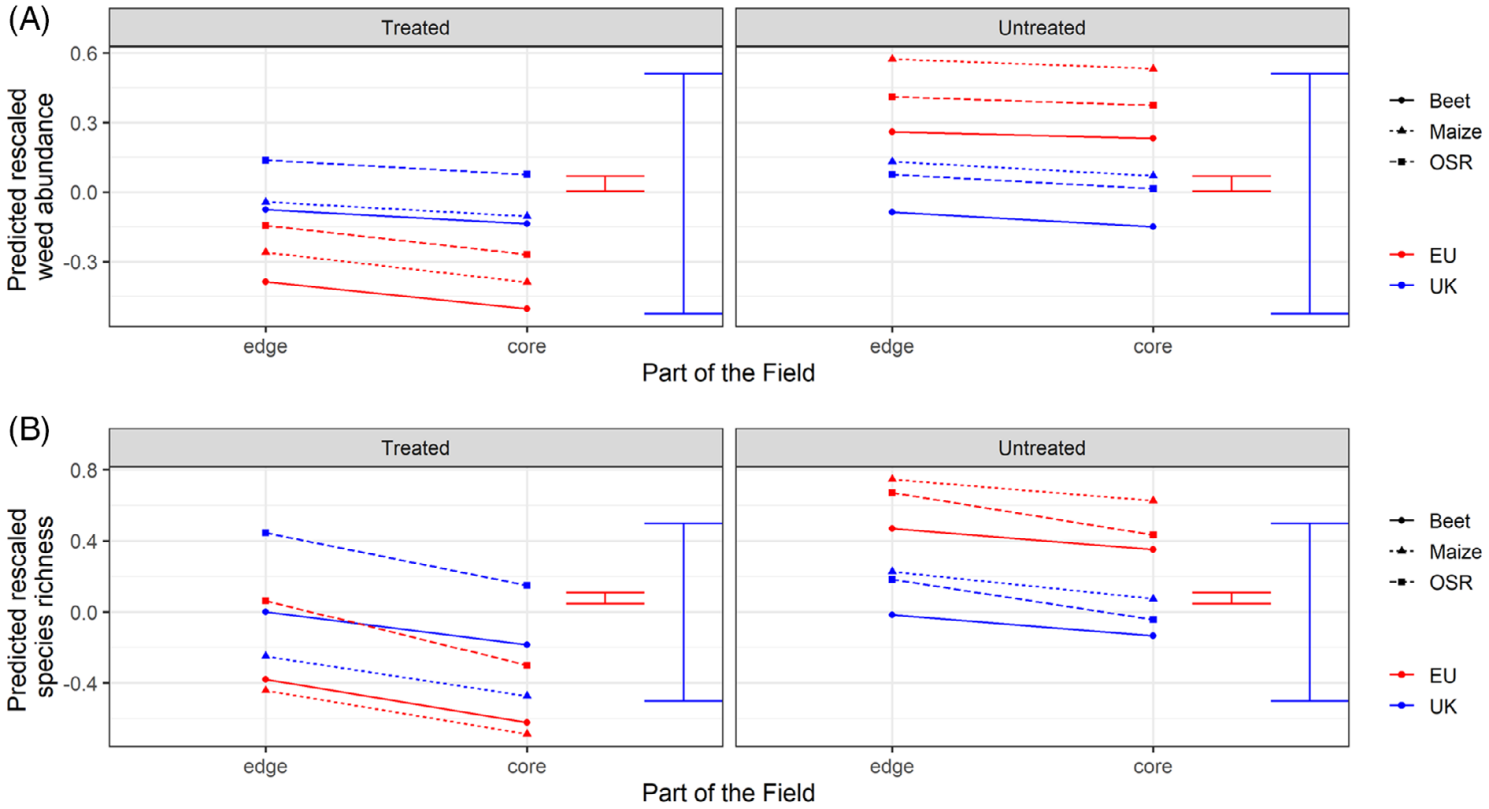
Predicted (A) weed abundance and (B) species richness (data rescaled to zero mean and unit variance) from a generalised linear mixed effects model. Predictions are classified by position in field, crop type (only three crops are shown here to avoid crowding the graphic), and herbicide treatment (treated/untreated). Results of step two of our analysis are shown in blue (UK) and step three (EU) in red. Error bars show the approximate average standard error of difference.

In our UK case study, both species richness and abundance were consistently higher at the field edge than in the field core (Figure 4). We also consistently find a strong effect of crop on both species richness and abundance (Table 4). Herbicide treatment was only found to have a significant effect at the European scale.

**TABLE 4:** Final generalised linear mixed model terms and their significance for both abundance and species richness at all three stages of the iterative analysis

Fixed effect	Step 1. UK (counts)	*N* = 24 432	Step 2. UK (rescaled to zero mean and unit variance)	*N* = 24 432	Step 3. Europe (rescaled to zero mean and unit variance)	*N* = 58 150
	Species richness	Abundance	Species richness	Abundance	Species richness	Abundance
Position in field (P)	<0.001***	<0.001***	<0.001***	<0.001***	<0.001***	<0.001***
Crop (C)	<0.001***	<0.001***	<0.001***	<0.001***	<0.001***	<0.001***
Herbicide treatment (H)	0.903^a^	0.949^a^	0.994^a^	1.000^a^	<0.001***	<0.001***
P × C			0.004**		<0.001***	<0.001***
P × H	0.072^a^		0.038*		<0.001***	0.006**
C × H	<0.001***	<0.001***	<0.001***	<0.001***	<0.001***	<0.001***
P × C × H						

*Note*: A separate model was fitted to each dataset for each response variable. *N* = The number of data points included in each dataset.

^a^ Term included in model but not significant, *p ≤ 0.05; **p ≤ 0.01; ***p ≤ 0.001, blank cells represent terms that were dropped from the model during the backwards selection process.

For all three case studies, despite the rescoping of the questions to include fewer variables with decreased resolution, the first step of the analysis yielded similar results to those seen in the original publications.

The transformation of response variables in step two of our analysis caused little change to the results observed. For example, switching from an analysis based on abundance to one based on presence/absence data in the FR case study gave a reduction in the explained inertia from 7.2% to 6.6%, but the relative importance of the explanatory variables was almost identical (Table 2, Spearman's rank correlation coefficient, *r* = 0.830, *p* = 0.006). Similarly in DE, where the resolution of the species data was reduced from count data to the 6‐level Barralis scale, the structure of variation partitioning stayed the same and the values remained similar.

In Step 3 of the analysis where we scaled up from national to European scale, we observed several changes in the results of the analyses. There was a gain in explained inertia (FR), a higher rate of explained variance (DE) and narrower confidence intervals on predictions (UK). Whilst the order of importance of variables tended to remain similar to the previous steps in all three case studies there were some notable changes in this final step, primarily in the geographic and climatic variables. In the FR case study, the main difference between the two spatial scales was the relative importance of soil and climatic variables. At the scale of France, soil pH was the first environmental variable, followed by precipitation, with less importance of longitude, latitude and temperature. Yet at the scale of Europe, longitude was the second most important variable immediately after crop type, and the influence of temperature was stronger at this scale. The effect of year was also stronger at the European scale. Similarly, in the DE case study, the combination of climate and geographical location explained most of the variation due to the environment at the European scale, whilst the net effects of climate, geographical location and soil were relatively small at the scale of Germany.

## 4 DISCUSSION

The consistency in results between the original studies and those we obtained in step one of our analyses indicates that despite the consolidation of several environmental and management variables and an associated reduction in the resolution of the data, this has little impact on the ability to answer important ecological questions using the data stored within AWME. In all three case studies, we were able to alter the scope of the question slightly to allow the inclusion of additional data sets from across Europe.

We identified several key challenges associated with combining data from multiple surveys. Some of these challenges are addressed within the AWME database itself, but for others it was necessary to address them in our analysis (Table 1). Through our three case studies, we highlighted some exemplary solutions to the challenges of combining disparate datasets. There is inherent data loss when analysing data from multiple sources as not all information is available from each source. In the case studies, we found that the resolution of explanatory variables was often coarser than in the original studies. There was also information loss from the exclusion of incomparable records or in the transformation of response variables (e.g., from counts to presence/absence). We addressed the challenge of different species abundance metrics by transforming the data to a common scale, which is known to influence the outcome of ordination analyses (Otypková & Chytry, 2006). However, we found that this transformation had little influence in our analysis. In case studies FR and DE, there was minimal impact on the percentage of explained inertia, and in case study UK, the variables identified as significant in the GLMM remained similar in each step of the analysis. This may be explained by the fact that the original datasets from France, Germany and the UK, were collected on a national scale representing a long gradient of ‘heterogeneous’ data with almost unique species composition in each plot. In this case, presence‐absence data is sufficient to describe between‐site variation. Whereas, in the case of more homogeneous data at the scale of a small region where many species are distributed in most sites, species abundance becomes the main source of between‐site variation and switching to presence‐absence data will have a more serious impact (Austin & Greig‐Smith, 1968). These findings suggest that future monitoring approaches aiming at analysis of species composition could focus on achieving a large sample size and reduce the sampling effort on each plot by estimating species abundance rather than counting individuals. This could allow a broader range of environments and/or management practices to be considered for the same sampling effort.

It is interesting to note that whilst the trend in the results observed for the UK dataset and the European dataset were similar in our UK case study, the magnitude of the abundance and species richness metrics diverged between the datasets. For both metrics, the absolute values of predictions for herbicide‐treated and untreated plots in the UK were very similar whilst at the European scale values were high in the untreated plots and much lower in the plots which had received herbicide. This exemplifies some of the key challenges described in Table 1. For the UK data, the herbicide‐treated and untreated data come from the same plots which were sampled before and after herbicide treatment and so we would expect the weed communities to remain similar with a loss of some individuals and species following treatment. However, in the rest of the AWME database the treated and untreated plots may come from vastly different surveys with different plot sizes and or different methodologies. It is also likely that the choice of the data collector to conduct a survey with or without herbicide treatment reflects the typical agronomy of the field, farm or region being surveyed. As such, survey data from sites with low or no herbicide use could be expected to have higher weed species richness and abundance than those where herbicide use is common (Hyvönen & Salonen, 2002). The choice to rescale the data within each dataset led to information loss on the absolute size of effect, however, it allowed us to consider all datasets with information on our variables of interest and the step‐wise analysis confirmed that this technique was effective in allowing us to understand the relative effects of our explanatory variables.

In all three case studies, we found some consistency in the conclusions that could be drawn at the national scale and at the European scale, particularly in terms of the role of management practices. In fact in all three cases, we saw an increase in the certainty of predictions or in the explanatory power of the fitted model. This is primarily due to the additional data incorporated into the analysis, but it also indicates that the patterns observed at the national scale are largely supported by the additional datasets as any contradictory data would likely weaken the strength of the results.

Where we observed differences in the results between the national scale and the continental scale was largely in the role of the environment. In the FR case study, crop management variables were the key drivers of weed community composition at the national scale with soil pH and precipitation being the most important environmental drivers. This result contrasted with other studies that indicated a stronger effect of environmental gradients (e.g., Lososová et al., 2004; Šilc & Čarni, 2007). However, when we repeated the analysis at the European scale the effect of longitude was much more important (second most important explanatory variable). However, even at this continental scale, the main difference between weed communities was still determined by the crop type, meaning there are more similarities between weed communities of a given crop in different European regions than between weed communities of two different crops within the same region. In France, a complex gradient of soil pH, precipitation and longitude opposed weed vegetation of dry calcareous regions in Eastern France to weed vegetation of more rainy acidic regions of Western France (Fried et al., 2008). Soil pH is recognised as one of the most structuring factors clearly differentiating acidophilic and basophilic weed assemblages (Hüppe & Hofmeister, 1990; Pinke et al., 2010). The soil effect seems less visible at the European scale may be due to a stronger differentiation of weed communities along longitudinal and temperature gradients.

Interestingly, in the FR case study, sampling season was the second most important gross effect at both the national and European scale. Therefore, the importance of the crop type variable is partly supported by warm continental countries (Pannonian plain of Hungary) or Mediterranean countries (Italy, Southern France) where the growing season is long enough to grow summer crops (Čarni et al., 2011). In northern Europe, the growing season is shorter and the differences between weed communities of winter and spring crops are less important. This difference could explain previous discrepancies between studies when reporting the relative importance of management (Hallgren et al., 1999) versus environmental factors (Lososová et al., 2004).

Beyond the case studies presented here, the large geographical extent covered by AWME makes it a particularly valuable resource to address questions where (i) a sufficiently large environmental gradient may not be present in national or regional surveys, like macroecological patterns or climate change effect prediction (space‐for‐time substitution) or (ii) an effect may be hypothesised to change with scale. Potential future studies using AWME could (a) test the abundant‐centre hypothesis (Sagarin & Gaines, 2002) which assumes that a species becomes more abundant at the centre of its range, where the environmental conditions are most favourable, (b) test the theory of species assembly (Booth & Swanton, 2002) according to hierarchical filters starting from a true regional pool, (c) predict responses to climate change and/or extreme events using a time‐for‐space substitution, (d) predicting the spread of invasive or troublesome weeds by identifying combinations of management practices, soil and climates suitable for these species to establish, (e) disentangling the effects of management systems and single management measures and (f) examining relationships between weed management, weed abundance/weed pressure and weed diversity.

We have demonstrated the utility of gathering weed survey datasets into a European‐scale database and explored the challenges and opportunities that such a database presents. We demonstrated that the European scale allows us to confirm and enrich previous works. Our case study results should encourage us to use existing datasets to tackle more ambitious issues which require the perspective of a larger geographical area or a range of spatial scales as these can be combined with minimal loss of information despite different methodologies. The AWME database is a growing collection, and we welcome new data contributions and requests for data for analysis.

## CONFLICT OF INTEREST

The authors have no conflicts of interest to declare.

### PEER REVIEW

The peer review history for this article is available at https://publons.com/publon/10.1111/wre.12562.

## Supporting information


**Appendix S1:** Supporting Information

## Data Availability

The data within AWME are available on the submission of a proposal to the coordination group. Data from individual datasets will be released according to their corresponding availability regime with the majority of data accessible for all research needs. Further information is available at https://ewrs.org/en/info/Blog/93/Arable‐Weeds‐and‐Management‐in‐Europe‐AWME‐A‐database‐of‐weed‐survey‐data‐from‐across‐Europe.
