# Supplementary material for: The utility of the ‘Arable Weeds and Management in Europe’ database: Challenges and opportunities of combining weed survey data at a European scale
Source: Weed Res. 2022 Dec 8;63(1):1–11. doi: 10.1111/wre.12562 (PMC10108295; doi:10.1111/wre.12562)
Supplement: Supplementary file 1 — Appendix S1: Supporting Information [file WRE-63-1-s001.docx]

**TABLE S1** Details of the AWME contributed datasets

| dataset | publication | Extent of study | Country/ Region | Year of survey | Plot size | Field parts | Number of observations in dataset | Number of fields ( if >1 observation per field) | Number of records in AWME | Abundance estimate |
| --- | --- | --- | --- | --- | --- | --- | --- | --- | --- | --- |
| CZ_01 & CZ_02 | Kolářová et al. (2013) DOI: 10.1111/wre.12045 | national | Czech Republic | 2006-2008 | 100 m² | Core | 290 |  | 132 + 156 | modified Braun-Blanquet |
| DE_01 | von Redwitz & Gerowitt (2018) DOI: 10.1111/avsc.12384 | regional | Germany | 2011-2013 | 10 * 0.1 m² pooled | Core | 255 | 224 | 255 | density |
| DE_02 | De Mol et al. (2015) DOI:10.1111/wre.12169 | national | Germany | 2000-2004/ 2008-2009 | 10 * 0.1 m² pooled | Core | 2756 |  | 2,881 | density |
| DE_03 | Hanzlik et al. (2011) DOI:10.1016/j.agee.2011.03.010 | national | Germany | 2005-2007 | 10 * 0.1 m² pooled | Core | 1,463 |  | 1,461 | density |
| DE_04 | Ulber et al. (2009) DOI:10.1111/j.1365-3180.2009.00722.x | regional | Germany | 2007 | 100 m² | Core & Edge | 96 | 24 | 94 | modified Londo |
| DE_07 | Glemnnitz (unpublished) | regional | Germany | 2000 - 2015 | 25m² | Core | 2,955 | 43 | 2,798 | modified Braun-Blanquet |
| DE_08 | Schumacher et al. (2018) DOI:10.5073/jka.2018.458.004 | regional | Germany | 2016 | 100 m² | Core & Edge | 28 | 14 | 28 | cover |
| DE_09 & DE_10 | Schumacher et al. (2018), DOI:10.3390/agriculture8110172 | regional | Germany | 2017, 2018 | 100 m² | Core & Edge | 96 + 58 |  | 96 + 58 | extended Braun-Blanquet |
| DE_11 | Schumacher (unpublished) | regional | Germany | 2015-2017 | 60 m² | Core | 100 | 25 | 23 | extended Braun-Blanquet |
| EU_01 | Glemnitz et al. (2006) Journal of Plant Diseases and Protection Special Issue XX, 577-586 | regional/ transnational | Germany, Hungary, Italy, Finland, Sweden, | 1999 - 2003 | 0.5-1 ha | Core & Edge | 266 |  | 70 | cover |
| EU_02 | Glemnitz et al. (unpublished) | regional/ transnational | Germany, Hungary, Italy | 2004 | NA | Core | 57 |  | 43 | frequency/ cover |
| FR_01 & FR_03 | Fried et al. (2008) DOI:10.1016/j.agee.2008.05.003 | national | France | 2002-2010 | 2000 m² /140m² | Core | 8 417 + 9242 | 1,396 | 8 401 + 9 223 | Barralis |
| FR_02 | Quinio et al. (2017) DOI:10.1016/j.eja.2016.10.011 | local | France | 2006-2012 | 2000 m² | Core | 152 | 136 | 132 | Barralis |
| HU_01 | Pinke et al. (2018) DOI:10.1016/j.cropro.2017.06.018 | national | Hungary | 2015-2016 | 50 m² | Core & Edge | 720 | 180 | 360 | cover |
| HU_02 | Pinke et al. (2011) DOI: 10.1111/j.1365-3180.2011.00885.x | national | Hungary | 2010 | 50 m² | Core & Edge | 408 | 102 | 204 | cover |
| HU_03 | Pinke et al. (2016) DOI: 10.1111/wre.12225 | national | Hungary | 2013 - 2015 | 50 m² | Core & Edge | 1048 | 262 | 524 | cover |
| HU_04 | Pinke et al. (2012) DOI:10.1111/j.1654-109X.2011.01158.x | national | Hungary | 2009 | 50 m² | Core & Edge | 972 | 243 | 486 | density |
| IT_01 & IT_02 | Vidotto et al. (2016) DOI:10.1016/j.eja.2015.11.018 | regional | Italy | 1999-2005 | 4 * 0.25m pooled | Core | 841 + 217 | 303 | 841 + 171 | density & cover |
| IT_03 | Vidotto and Fogliatto (unpublished) | regional | Italy | 1996-2000 | 4 * 0.25m pooled | Core | 317 | 130 | 230 | density & cover |
| IT_04 & IT_05 | Vidotto and Fogliatto (unpublished) | regional | Italy | 2008-2012 | 4 * 0.25m pooled | Core | 112 | 56 | 42 + 55 |  |
| LV_01 | Ņečajeva (unpublished) | national | Latvia | 2017 | 100 * 0.2 m² pooled | Core | 293 | 51 farms | 287 | Rasins & Taurina |
| LV_02 | Ņečajeva et al. (2015) DOI:10.17770/etr2015vol2.275 | regional | Latvia | 2013 - 2014 | 100 * 0.2 m² pooled | Core | 600 | 51 farms | 585 | Rasins & Taurina |
| PL_01 | Dostatny D. F., (unpublished) | regional | Poland | 1997-1999 | 100 m² | Edge | 293 |  | 293 | Braun-Blanquet |
| SP_01 | Hernández Plaza and González-Andújar (unpublished) | regional | Spain | 2014 | 1m² | Core | 150 | 15 | 15 | cover |
| SP_02 | Hernández Plaza and González-Andújar (unpublished) | regional | Spain | 2017 | 1m² | Core | 180 | 18 | 18 | density |
| SP_03 | Hernández Plaza and González-Andújar (unpublished) | regional | Spain | 2018 | 1m² | Core | 120 | 12 | 12 | density |
| SP_04 | Final carreer project: open access, but in Catalan with English abstract | regional | Spain | 2015 | 4-10 * 0.1 m² pooled | Core | 66 |  | 65 | density |
| SP_05 | Cirujeda et al. (2019) DOI:10.3390/agronomy9030134 | regional | Spain | 2011 - 2014 | 1 ha (zigzag transects) | Core | 120 |  | 120 | CEB scale |
| SP_06 | Cirujeda et al. (2011) DOI:10.1007/s13593-011-0030-4 | regional | Spain | 2005-2007 | 2 ha (zigzag transects) | Core | 138 |  | 138 | CEB scale |
| UK_01 & UK_02 | Firbank et al. (2003) DOI:10.1046/j.1365-2664.2003.00787.x | national | UK | 2000-2002 | 0.125 m² | Core | 12 866 + 12 016 | 270 | 520 + 511 | density |

**TABLE S2** Summary statistics of continuous environmental variables contained within the AWME database demonstrating the length of gradients covered

| **Variable** | **Min** | **Median** | **Mean** | **Max** |
| --- | --- | --- | --- | --- |
| Latitude (°N) | 36.4 | 51.7 | 50.3 | 70.4 |
| Longitude (°E) | -6.3 | 0.9 | 3.4 | 31.1 |
| Mean Temperature (°C) | 0.5 | 9.6 | 9.9 | 18.2 |
| Minimum Temperature (°C) | -17.8 | 0.0 | -0.1 | 7.8 |
| Precipitation (mm) | 358.0 | 681.0 | 699.6 | 1399.0 |
| Summer Precipitation(mm) | 17.0 | 172.0 | 181.1 | 378.0 |

**Supplementary Methods**

**Spatial subsampling in Case study 2**

The number of plots with complete data of the four datasets was of very different sizes (DE1 238, DE2 2355, FR1 9, FR3 753) and the spatial sampling density varied. Therefore, we tested a spatial subsampling procedure using grids with increasing width (from 0.1° to 3°). The spatial extent of the European dataset was divided in cells of equal size and one record per cell chosen at random. With this subsample, we performed variation partitioning and extracted the explained inertia. This process was repeated 200 times for every grid width.

The subsampling procedure of the dataset came along with increasing variance for larger grid sizes which led to smaller subsample sizes. At grid sizes of 0.1 to 0.5° the difference between a thinned-out dataset to the complete dataset was negligible but starting at 1.0° the results lost quality. Additionally, we could show that the single datasets which were originally contributed to AWME had a smaller explanation strength then the combined dataset.

Our investigations into grid-based subsampling (case study 2) further supports this conclusion. Smaller grid sizes gave a larger number of retained samples, which on average lead to lower variance. The analysis showed that results were most consistent when using all samples from the combined dataset and not a smaller subsample. However, the variation increased slowly, showing that global analyses do not need to use a fine-grained raster. We concluded that subsampling was not necessary in our case.


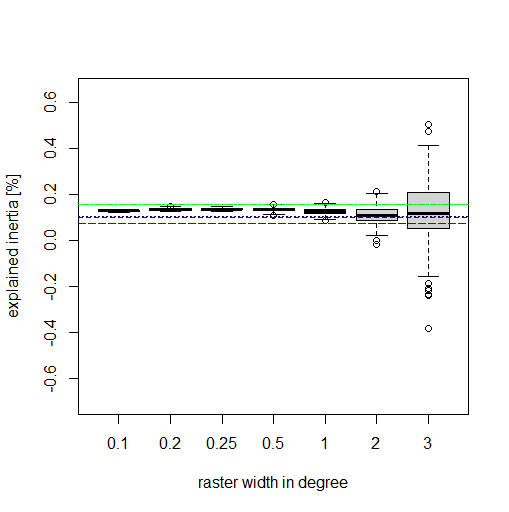


**FIGURE S1** Explained inertia of spatial subsampling with 200 repetitions on different grid widths. Horizontal lines are the explained inertia of the selected European dataset used in Case study 2 (green) and the originally contributed datasets from France (dark red) and Germany (blue).
